# Using prescribing and toxicology data to determine non-medical prescription drug overdose

**DOI:** 10.1016/j.abrep.2020.100289

**Published:** 2020-06-08

**Authors:** Philip Huynh, Grant Victor, Brad Ray

**Affiliations:** Center for Behavioral Health and Justice at Wayne State University, Detroit, MI, United States

**Keywords:** Overdose, Toxicology, PDMP, Opioids, Non-medical prescription drug use

## Abstract

**Background:**

Overdose deaths have increased dramatically in the United States and are often attributed to prescription opioids. This study presents a framework for “overdose typologies”, including non-medical prescription drug use, to more accurately describe drug use patterns.

**Methods:**

This study examined linked prescription drug monitoring program (PDMP) and toxicology data (2016–2018) from accidental overdose deaths from a large metropolitan coroner’s office in the Midwest (Indianapolis, Indiana).

**Results:**

In total, 1,112 accidental overdose deaths occurred and over two-thirds (68.0%; n = 756) were coded as an illicit drug user with no prescription opioid present in the toxicology. The most infrequent categories were prescription opioid users 5.5% (n = 61).

**Conclusion:**

Linked PDMP and toxicology reports are useful in identifying drug use patterns that contribute to mortality.

## Highlights

• Many fatal overdoses (68%) had illicit substances without any prescription opioids.
• Few fatal overdoses had the same prescription present that they were prescribed.
• Around half of all fatal overdoses had a corresponding prescription drug history.
• Prescription and toxicology data are useful in identifying drug use patterns.

## 1 Introduction

The United States is in the midst of an overdose crisis with more than a half million accidental drug overdose deaths having occurred since 2000, and over 70,000 deaths in 2017 alone (Scholl, 2019). While the majority of these deaths are associated with opioids, the specific type of opioid has varied dramatically across three waves (Ciccarone, 2019), shifting from prescription medications, to heroin, and then fentanyl, with each wave resulting in increased mortality rates. However, the over prescription of opioid analgesics, which is often attributed to the adoption of pain as a vital sign, is consistently considered a contributing factor in the overdose epidemic (Rummans, Burton, & Dawson, 2018). One widespread policy response to the overdose crisis has been adopting and expanding the use of state-wide surveillance systems to monitor and detect the prescription of opioids. Referred to as prescription drug monitoring programs (PDMPs), these systems record controlled substances that were dispensed and/or prescribed within their respective state boundaries.

Patient PDMP records have been used as a reference tool to help prescribers and pharmacists identify ‘high risk’ patients and by law enforcement officials to identify diversion and doctor shopping (Hopkins, Holt, & O’Leary, 2014). As such, PDMP legal reforms focus on a number of areas such as the defining agencies that have access to information, specifying purposes for which the information can be used, if access can be delegated, and whether patients themselves can access to their data (Centers for Medicare and Medicaid Services, 2017, Substance Abuse and Mental Health Services Administration, 2017). While much consideration has been given to whether law enforcement agencies should have access to PDMP patient data (Catalini, 2017, Islam and McRae, 2014, Robeznieks, 2017) there has been less focus on policies around other agencies that might access this information. For example, the Prescription Drug and Abuse Policy System (PDAPS) reports that 31 jurisdictions have provided access to PDMPs for medical examiners and coroners for the purposes of investigating cause of death (Science, 2016). Under this configuration, PDMP records can be obtained by the coroner to determine which substances were prescribed.

Several studies have linked PDMP data with toxicology reports to find many overdoses involved a prescription opioid; although, many of these studies pre-dated the increased market prevalence of heroin and illicit fentanyl. For instance, studies conducted with data from North Carolina, Tennessee, and Kentucky found that between 60% and 80% of opioid overdose decedents had an opioid prescription, respectively (Austin et al., 2017, Nechuta et al., 2018, Slavova et al., 2017). In North Carolina, one study found 47% of decedents had an active prescription for oxycodone in the 30-days prior to death (Austin et al., 2017); in Kentucky, found that 33% of decedents had an active opioid prescription at the time of death (Slavova et al., 2017); and in Tennessee found that an active prescription opioid was detected in 55% of prescription opioid overdoses, 39.2% of fentanyl overdoses, and 20.7% of heroin overdoses (Nechuta et al., 2018). However, a recent study from Massachusetts looked to see whether the specific prescription opioids found in the toxicology results from an overdose death matched a prescription to reveal that only 1.3% had an active prescription for the opioid involved in the death (Walley et al., 2019).

In the present study we further extend on the utility of PDMP access among coroners by linking three years (2016–2018) of PDMP and toxicology data from accidental overdose deaths from a large metropolitan coroner’s office. Our intent was to develop a framework for identifying non-medical prescription drug use, an area of substance use and overdose that is not well-defined (Green, Black, Serrano, Budman, & Butler, 2011), and use these data to develop “overdose typologies” that more accurately describe drug use patterns. Federal and state efforts have focused on improving and integrating overdose data to provide more timely surveillance on a rapidly ever-changing public health crisis (Centers for Disease Control & Prevention, 2019); creating typologies can help to determine drug use prevalence patterns and how public health resources can be allocated to reduce overdose deaths. Prior research has indicated, the non-medical use of prescription opioids can be a significant predictor in developing a substance use disorder issue especially if non-medical use occurs early in an individual’s life (Compton and Volkow, 2006, Ford and Watkins, 2012, McCabe et al., 2007). By integrating the PDMP into the toxicology results we were able to identify those cases where prescription medications played a role in the death but also when those prescriptions were, and were not, legally prescribed around the time of death. In doing so, we can better assess the role of nonmedical opioid and non-opioid prescription drug misuse in fatal overdose deaths

## 2 Study data and methods

The current study uses data from Marion County, Indiana (Indianapolis), where in 2016 state legislation was passed that allowed coroners to examine information from the Indiana PDMP, *Indiana Scheduled Prescription Electronic Collection and Tracking* (INSPECT). Marion County is the largest county in the state, home to Indiana’s capital of Indianapolis, but also where nearly one-quarter of all fatal overdose deaths occur in the state. As part of a CDC-funded study, the Marion County Coroner’s Office (MCCO) had partnered with local researchers to collect toxicology and death certificates on all accidental drug overdose deaths (X40-X44) since 2010 (Lowder et al., 2018, Phalen et al., 2018, Ray et al., 2017). Following the 2016 legislation the MCCO Deputy Coroner developed procedures to amend each overdose death with a PDMP report from INSPECT. This included searching the INSPECT system for a decedent’s entire prescription history a year prior to their death using their full name, date of birth, and other demographic information if necessary. Any prescription that was filled in the year prior to the decedent’s death was included in the study population. At the time of this study, time frames of prescription fill dates with the decedent’s date of death were not used in the analysis.

Data from death certificates provided sociodemographic information while toxicology data provided detection (which is based on thresholds set by the testing agency) on the all substances present at the time of death. During the three-year time span of the study, more than 90% of all toxicology testing was completed by a single agency and the remaining cases were sent to a separate toxicology vendor. Regardless of the agency, all results adhered to the reporting requirements set by each agency and each toxicology screen was individually interpreted by the coroner.

For the current study, we focused only on a subset of illicit and prescription substances available from the toxicology results. Illicit substances included 6-monoacetylmorphine (heroin), fentanyl (and synthetic analogues such as carfentanyl), methamphetamine, and cocaine. Prescription substances of interest included prescription opioids (such as oxycodone, hydrocodone, oxymorphone, hydromorphone, and tramadol) and benzodiazepines. The linked PDMP data included information on the type of controlled substance prescribed, quantity, dose, MME (morphine milligram equivalents) if applicable, written and filled dates, and prescriber and dispenser information.

**Fig. 1:**
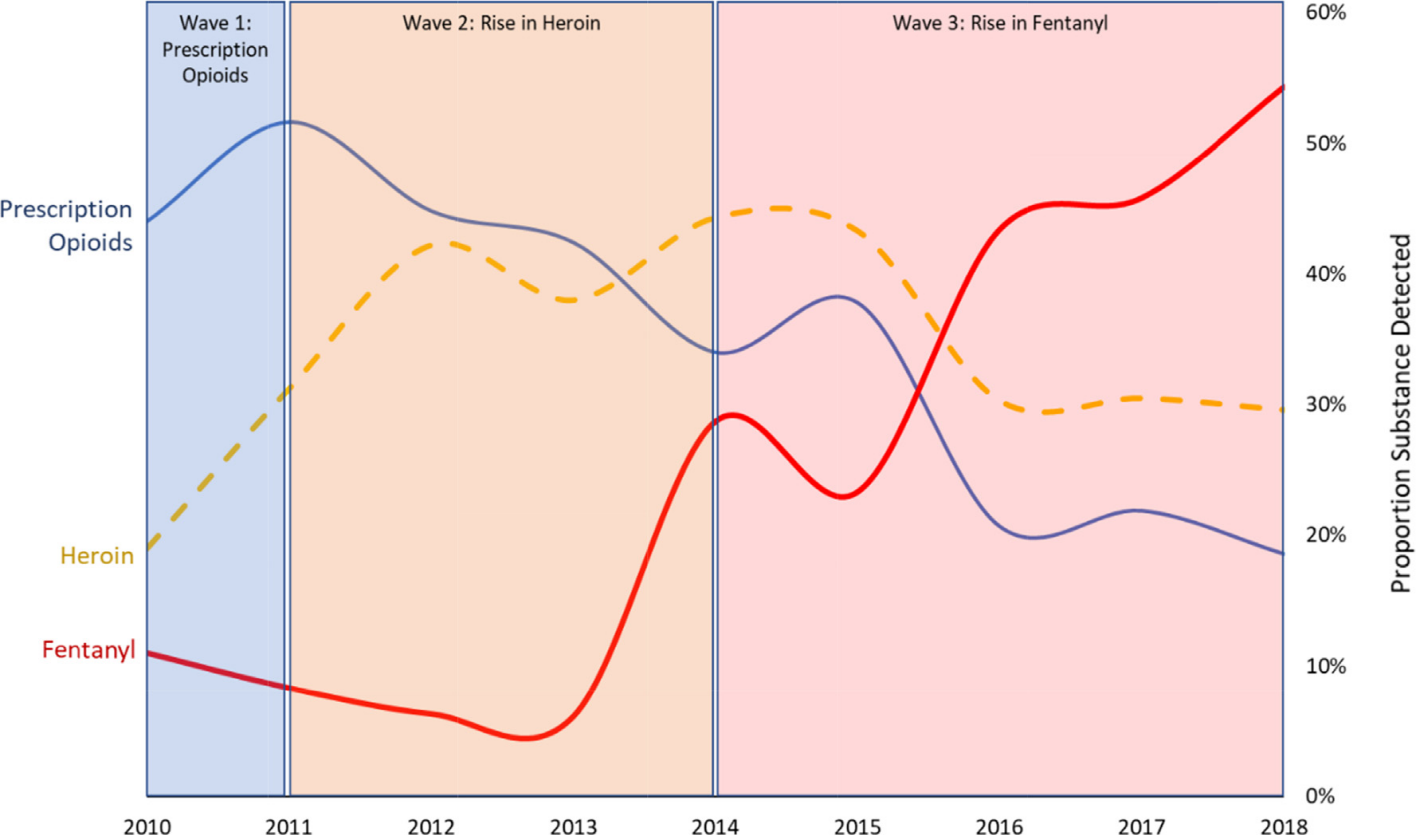
Waves of the opioid epidemic.

Death certificates and toxicology reports have been collected from the MCCO since 2010 and comprises of 2336 accidental drug overdose deaths as of December 31, 2018. Throughout this nine-year time period, trends in the types of substances followed a pattern which began with prescription opioids, moving to heroin in 2011, then fentanyl in 2013 (Fig. 1). This is consistent with national trends that found that the opioid epidemic occurred with similar “waves” (Centers for Disease Control Prevention, 2018).

## 3 Overdose typologies

We used both the toxicology and PDMP data to create five mutually exclusive categories of drug users. First, cases where there was a match between the prescription opioids detected in the toxicology results and the PDMP data were coded as prescription opioid drug-related death. Regarding prescription opioids, the following substances were examined: hydrocodone, hydromorphone, oxycodone, oxymorphone, methadone, tramadol, morphine, and codeine. When there was no match with the above substances in the PDMP data, but these substances were found in the toxicology report, the case was coded as a nonmedical prescription opioid drug-related death. Thus, we assume that prescription medications were either obtained through nonmedical sources or taken outside of the prescribing period. Next, we created a separate illicit drug code based on whether the toxicology data included 6-monoacetylmorphine, fentanyl, cocaine, or methamphetamine. We then interacted these categories to create the following five classifications:

Opioid prescription with illicit drug detection

• Prescribed opioid(s) with prescription opioids and illicit drugs present in toxicology report

Opioid prescription without illicit drugs detection

• Prescribed opioids and does not have prescription opioids and illicit drugs in toxicology

Nonmedical opioid prescription with illicit drug detection

• Has prescription opioid in toxicology without being prescribed opioids and has illicit drugs in toxicology

Nonmedical opioid prescription without illicit drugs detection

• Has prescription opioid in toxicology without being prescribed opioids and does not have illicit drugs in toxicology

Illicit drugs with no prescription opioids

• Has illicit drugs in toxicology but no prescription opioids in toxicology

Our analysis focused on examining differences between those cases where a PDMP record was detected but also using the linked toxicology data to develop overdose typologies. Descriptive and correlation statistics were used to examine and describe the results. Statistical tests that were performed include t-tests and the level of significance was α = 0.05, with all *p*-values lower than that value considered statistically significant.

## 4 Study results

Over a three-year period (January 2016 – December 2018) there were 1112 accidental overdose deaths in Marion County, Indiana. Among them, 47.5% (n = 528) had a corresponding PDMP patient report. Of these, over three-quarters (n = 412, 78.0%) had a prescription for an opioid in the year prior to their death, and about half (n = 266, 50.4%) had non-opioid prescriptions (i.e., benzodiazepines, amphetamines, anticonvulsants, barbiturates, muscle relaxants, or sedatives) (Table 1). Table 1 also shows the frequency in which a decedent’s prescription was the same substance which was found in their system at the time of death. Overall, among individuals prescribed prescription opioids (n = 412), 34.2% (n = 141) had a prescription opioid detected in their toxicology analysis. Among descendants with a non-opioid prescription, 177 (66.5%) also had a non-opioid prescription in their system.

**Table 1:** Prescribed Substances Detected in Toxicology (n = 528).

	PDMP	Toxicology	Matches*
**Prescription Opioids**	412 (78.0)	160 (30.3)	141 (34.2)
Morphine*	20 (3.8)	85 (16.1)	6 (30.0)
Codeine*	44 (8.3)	1 (2.3)	1 (2.3)
Hydrocodone	316 (59.8)	68 (12.9)	58 (18.4)
Hydromorphone	5 (0.9)	41 (7.8)	3 (60.0)
Methadone	6 (1.1)	22 (4.2)	5 (83.3)
Oxycodone	156 (29.5)	56 (10.6)	42 (26.9)
Oxymorphone	5 (0.9)	36 (6.8)	4 (80.0)
Tramadol	60 (11.4)	14 (2.7)	9 (15.0)

**Non-Opioid Prescriptions**	266 (50.4)	316 (59.8)	177 (66.5)
Benzodiazepine	215 (40.7)	221 (41.9)	119 (55.3)
Amphetamine***	37 (7.0)	131 (24.8)	15 (40.5)
Anticonvulsant	41 (7.8)	20 (3.8)	4 (9.8)
Barbiturate	11 (2.1)	5 (0.9)	3 (27.3)
Muscle Relaxant	12 (2.3)	26 (4.9)	4 (33.3)
Sedative/Hypnotic	32 (6.1)	4 (0.8)	2 (6.3)

**Illicit Substances**			
Fentanyl***	8 (1.5)	226 (42.8)	6 (75.0)
6-monoacetylmorphine (Heroin)	Not applicable	171 (32.4)	Not applicable
Cocaine	Not applicable	122 (23.1)	Not applicable
Methamphetamine	Not applicable	131 (24.8)	Not applicable

*Morphine and codeine not counted if heroin was present in toxicology screen.

**Each match is not mutually exclusive from each substance and percent based off PDMP prescription.

***Cannot differentiate between prescribed and illicit fentanyl or amphetamine in toxicology.

Looking at cases who had an PDMP record (n = 528), decedents who had an opioid prescription (n = 412) had an average of 11.7 (SD = 12.7; Range 1–64) prescriptions in the year prior to death with an average of 3.2 (SD = 2.7; Range 1–19) providers and 2.7 (SD = 2.1; Range 1–14) dispensers. This was each statistically higher compared to decedents had only non-opioid prescriptions (n = 116) (prescriptions t(526) = 3.076, p = 0.002; providers t(526) = 7.908, p < 0.001; dispensers t(526) = 3.471, p = 0.001). Those who had only non-opioid prescriptions (n = 116) had an average of 8.6 (SD = 8.7; Range 1–44) prescriptions in the year prior to death with an average of 1.8 (SD = 1.2; Range 1–8) providers and 2.1 (SD = 1.4; Range 1–8) dispensers (Table 2). The most commonly prescribed opioid prior to death were hydrocodone, oxycodone, and tramadol while the most recently filled dispensations before the decedent’s death occurred on average 105 days (SD = 108.1; Range 1–365) prior to death. Nearly a third of all those prescribed any substances had an opioid dispensed to them less than 30-days before their death (34.3%, n = 143).

**Table 2:** Prescribing Characteristics.

	Overall (n = 528)		Opioid Rx (n = 412)		Non-Opioid Rx (n = 116)		*t*	*p*-value
Mean (S.D.) Range								
Prescriptions	11.0 (12.0)	1–64	11.7 (12.7)	1–64	8.6 (8.7)	1–44	3.076	0.002
Providers	2.9 (2.5)	1–19	3.2 (2.7)	1–19	1.8 (1.2)	1–8	7.908	<0.001
Dispensers	2.6 (2.0)	1–14	2.7 (2.1)	1–14	2.1 (1.4)	1–8	3.471	0.001

Table 3 shows the frequency of prescription opioid drug-related death, nonmedical prescription opioid drug-related death, illicit drug-related death and the overdose typology classifications. Over two-thirds of the cases (68.0%; n = 756) were coded as an illicit drug user with no prescription opioid present in the toxicology. It is important to note that among this category, 40.9% (n = 309) still had a prescription in the year prior to death. For further clarity, these cases were removed, and 40.2% (n = 447) of the overdose cases were exclusively illicit drug users. The next most common category was prescription opioid users with illicit drugs 7.2% (n = 80), followed by nonmedical prescription opioid users with illicit drugs at 6.3% (n = 70). The most infrequent categories were prescription opioid users without illicit drugs at 5.5% (n = 61) followed by nonmedical prescription opioid user without illicit drugs (i.e., cases where a prescription opioid was detected in the toxicology report but there was no detection of illicit substances and no prescription opioid PDMP match) at 1.4% (n = 16). There were also 129 cases (11.6%) where none of the substances used in this matching scheme were detected.

**Table 3:** Overdose Typologies.

Drug User Classifications	PDMP		
	No	Yes	Total
	(n = 584)	(n = 528)	(N = 1112)
**Prescription (Rx) Opioid User**	Not applicable	141 (34.2)	141 (12.7)
(1) Rx Opioid with Illicit Drugs	Not applicable	80 (15.2)	80 (7.2)
(2) Rx Opioid without Illicit Drugs	Not applicable	61 (11.6)	61 (5.5)

**Nonmedical Prescription Opioid User**	67 (11.5)	19 (3.6)	86 (7.7)
(3) Nonmedical Rx User with Illicit Drugs	54 (9.2)	16 (3.0)	70 (6.3)
(4) Nonmedical Rx User without Illicit Drugs	13 (2.2)	3 (0.1)	16 (1.4)

**Illicit Drug User**			
(5) Illicit Drug User with No Opioid Rx	447 (76.5)	309 (58.5)	756 (68.0)

No substances matched	70 (12.0)	59 (11.2)	129 (11.6)

## 5 Discussion

This is the first study to link PDMP and toxicology data to establish typologies, which includes nonmedical prescription drug use, among a population of drug-related decedents using this information. Findings suggest that among those with a PDMP record (N = 528; 47.5%), over a third (n = 141, 34.2%) were prescribed an opioid in the year prior to their death (n = 412, 78.0%) and had the corresponding opioid detected in their toxicology screen. In addition, the prevalence of matching prescribed non-opioid substances (i.e., benzodiazepines, anticonvulsants, sedatives, amphetamines, muscle relaxants, and barbiturates) present in toxicology screens among those prescribed a non-opioid drug (n = 266, 50.4%) was higher in comparison to those who were prescribed opioid prescriptions (n = 177, 66.5% vs. n = 141, 34.2%). Hydrocodone (n = 316, 59.8%) was the most commonly prescribed opioid among decedents with a detected prescription drug in the toxicology record. This may be because hydrocodone is commonly used as a short-acting outpatient analgesic (Krashin, Murinova, & Trescot, 2013). The second most frequently detected prescription opioid from toxicology was oxycodone (n = 56), which is very similar to hydrocodone, has been routinely prescribed for both acute and chronic pain conditions. Although morphine (n = 20) was prescribed in considerably fewer PDMP records, it was the most common prescription opioid medication that was detected in toxicology records; however, correctly identifying morphine in opioid-related overdose deaths is challenging because of the rapid metabolism of heroin into morphine (Bogusz et al., 1997, Gottås et al., 2013).

The current study highlights five distinct overdose typologies: (1) prescription opioid user with illicit drugs; (2) prescription opioid user without illicit drugs; (3) non-medical prescription opioid user with illicit drugs; (4) non-medical prescription opioid user without illicit drugs; and (5) illicit drug user with no opioid prescription. The results from the present study suggest that in Marion County, Indiana between 2016 and 2018 the majority of deaths were illicit drug users without an opioid prescription (n = 756, 68.9%). Moreover, among all overdose deaths (n = 1112), fewer than 13% occurred with a prescription opioid that was prescribed to the decedent within 12-months before their death. Our typologies are consistent with the research out of Massachusetts which found that those with active prescriptions for similar substances (oxycodone or tramadol) were also detected in about 20% of postmortem toxicology reports as well as illicit substances, specifically fentanyl and heroin, detected in 45.3% and 61.4% of all deaths respectively (Walley et al., 2019).

At the policy level initial investigations on the effectiveness of PDMPs suggested significant decreases in opioid prescribing which correlated with decreases in prescription opioid-related morbidity and mortality (Johnson et al., 2014, Rutkow et al., 2015, Strickler et al., 2019). However, more recent evidence suggests the implementation of PDMPs, which restricted legal access to prescription opioids, may be associated with greater initiation of heroin and illicit fentanyl (Bunting et al., 2020, Ciccarone, 2019, Seth et al., 2018; Victor, Walker, Cole, & Logan, 2017). By linking PDMP and toxicology data this study illustrates some potential benefit of this policy at the local level that would only be extended by examining at these patterns statewide and over a longer period of time.

Among the decedents where a prescription opioid was detected in the toxicology and there was a linked PDMP record detected, the opioid in the toxicology was the same as that prescribed more than 90% of the time. Comparing those with a PDMP record and those without, individuals with legally prescribed opioids were less likely to show evidence of drug diversions than those who did not have legally prescribed opioid prescriptions (3.6%, n = 19). Among all included prescription medications, benzodiazepines (n = 221) and amphetamines (n = 131), were the two prescription medications that were most prevalent in toxicology reports. These findings support rising concerns related to the increasing role of non-opioid prescription medications and illicit stimulants in drug-related mortality (Ellis et al., 2018, Gladden, 2017). In addition, a recent report found that deaths involving fentanyl and benzodiazepines have increased over the first half of 2018 (Gladden, 2019), yet isolated prescription opioid and benzodiazepine deaths have decreased over the same period.

There remain substantial gaps in the literature regarding more recent drug-related mortality and the role of non-opioid prescription medication. Co-occurring illicit fentanyl and methamphetamine deaths are rising as evidenced by increases in methamphetamine supply (Gladden, 2019) and in increases of reported methamphetamine use among a nationally representative sample of individuals seeking opioid treatment (Strickland, Havens, & Stoops, 2019). However, it is critical that legislatures are informed by findings that examine drug mortality beyond ICD codes and better utilize toxicology results. Initiatives such as the Enhanced State Opioid Overdose Surveillance (ESSO) program from the Centers for Disease Control and Preventions have funded 33 states to improve data collection efforts on opioid overdoses through the use early warning systems, linkage of data from coroners and medical examiners investigations, and dissemination of findings across states (Centers for Disease Control & Prevention, 2019). In doing so, the present study underscores how these toxicology data, alongside PDMP data, can help us to better understand the epidemiology of polysubstance use behaviors and trends in mortality.

## 6 Limitations

The current study had several noteworthy limitations. First, the classification protocols used in the toxicology reporting may not precisely detect drugs that contributed to death due to varying standards across jurisdictions and to protocol changes over time. The classification of specific opioids may be particularly sensitive to detection given the rapid metabolism of certain drugs (e.g., heroin-to-morphine) and the classification of fentanyl as a prescription drug. Second, opioid prescriptions that were obtained at pharmacies outside of Indiana were not captured in the PDMP records. Third, the analyses did not allow for causal inferences of the role of drug use in the cause of death. Fourth, we did not consider the filled dates of prescribed prescriptions to determine if they overlapped with the decedent’s date of death and only if they were prescribed within a year prior to their death. Lastly, generalizations are limited since these data were linked from databases of a single county but also limited to a time period in which illicit opioids were involved an a record number of accidental drug overdose deaths, particularly in the Midwest(O’Donnell, Halpin, Mattson, Goldberger, & Gladden, 2017).

## 7 Conclusions

The primary contributors to drug overdose mortality in Indiana during the study period were illicit fentanyl and heroin. The prevalence of deaths involving illicit fentanyl, benzodiazepines, amphetamines, cocaine, and methamphetamine underscore the need to better understand drug use behaviors related to drug combinations and contaminations. Relative to illicit drug use, prescription opioid use posed a considerably lesser overdose risk. These findings support the utility of PDMP policies in mitigating misuse of prescription opioids, yet concerns persist regarding the effect these policies may have in producing patterns of higher risk drug use and misuse. Linking PDMP data with toxicology data is a useful tool in generating novel knowledge about fatal drug use and this practice should continue to be utilized and expanded by community-based researchers, treatment providers, and policy-makers, where appropriate. Expanding outreach efforts to vulnerable population groups and reducing barriers to treatment such as harm reduction services, housing services, and evidence-based practices is recommended.

## CRediT authorship contribution statement

**Philip Huynh:** Data curation, Formal analysis. **Grant Victor:** Writing - Review & Editing. **Brad Ray:** Funding acquisition.

## Declaration of Competing Interest

The authors declare that they have no known competing financial interests or personal relationships that could have appeared to influence the work reported in this paper.
